# Supplementary material for: Advancements in Monte Carlo simulations with gMicroMC: reactive species build-up promotes radical-radical reactions at Flash dose rates
Source: ArXiv. 2025 Sep 3:arXiv:2509.03313v1. Preprint. [Version 1] (PMC12425020)
Supplement: Supplement 1 [file NIHPP2509.03313v1-supplement-1.pdf]

Supplementary material

		Chanel	Probability
**Ionization**	H_2O^+	$H^+ + {}^{\cdot}OH$	100
**Excitation**	A^1B_1	$H^{\cdot} + {}^{\cdot}OH$	65
		H_2O	35
	B^1A_1	$H^+ + {}^{\cdot}OH + e^-_{aq}$	50
		$H^{\cdot} + {}^{\cdot}OH$	25.35
		$H_2 + 2{}^{\cdot}OH$	3.25
		$2H^{\cdot} + O(^3P)$	3.9
		H_2O	17.5
	Rydberg, Diffusion Bands	$H^+ + {}^{\cdot}OH + e^-_{aq}$	50
		H_2O	50
**Electron Capture**	Electron Attachment	${}^{\cdot}OH + OH^- + H_2$	100
	Electron Hole	$H^{\cdot} + {}^{\cdot}OH$	35.75
		$H_2 + 2{}^{\cdot}OH$	13.65
		$2H^{\cdot} + O(^3P)$	15.6
		H_2O	35
**Electron Solvation**	Hydratation	e^-_{aq}	100

Table S1: Branching ratios and associated probability for each channel included in gMicroMC collected from Shin et al.[45].

**Chemical Species**	$\mathcal{D}$ **(10⁹·nm²s⁻¹)**
e^-_{aq}	4.82
${}^{\cdot}OH$	2.2
$H^{\cdot}$	7.0
H^+	9.3
H_2	4.8
OH^-	5.3
H_2O_2	2.3

Table S2: Chemical species and their respective $\mathcal{D}$ included in gMicroMC collected from Elliot et al.[6].

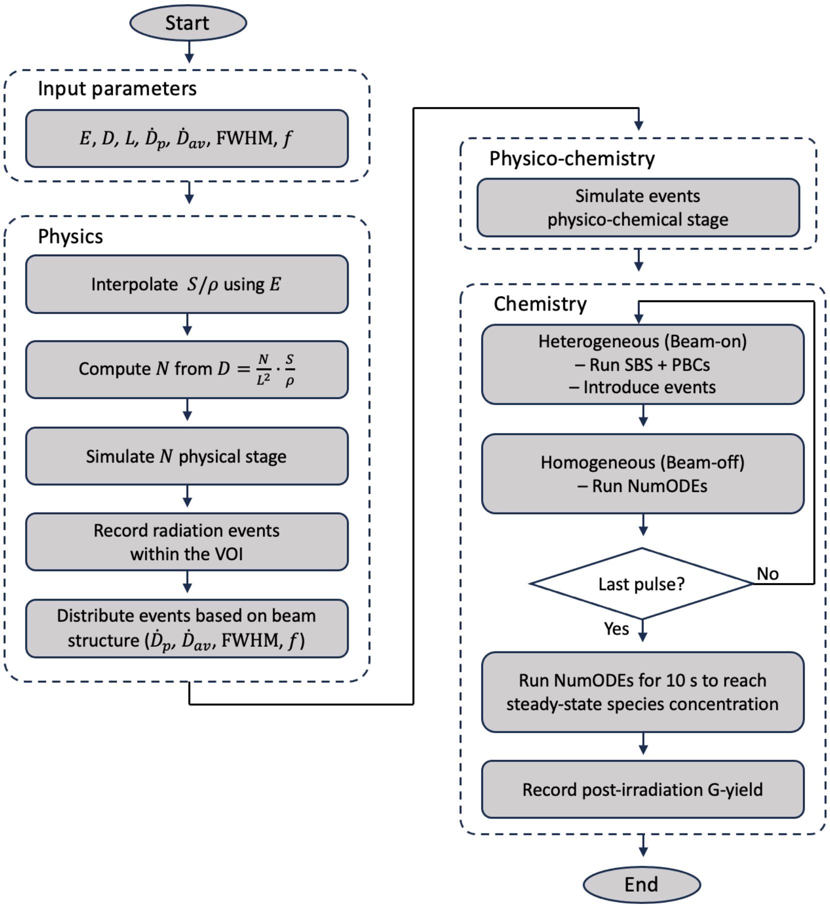

Figure S1: Flow chart depicting the gMicroMC extension for multiple pulse irradiations.

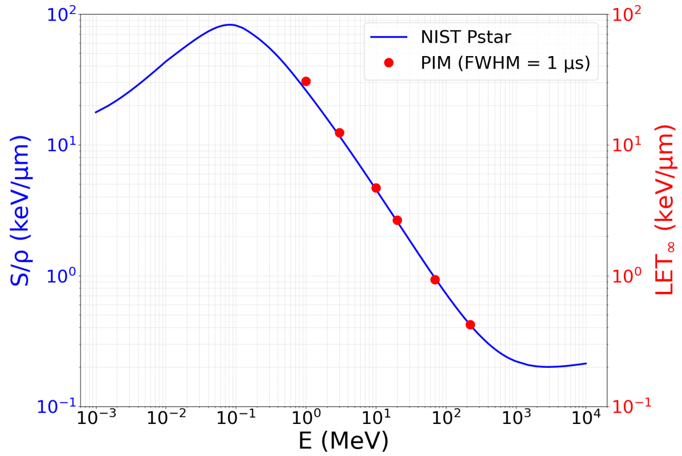

Figure S2: LET_∞ within a VOI characterized by $L = 5$ μm for single pulse radiolysis characterized by FWHM = 1 μs and $\dot{D}_p = 10^6$ Gy/s. It is shown the S/ρ obtained from NIST Pstar[37] for the corresponding E.

Reaction radius calculation for self-radical recombination reactions

The mathematical analysis for the calculation of the reaction radius (R) for diffusion controlled reactions is presented below, utilizing the Smoluchowski phenomenological approach[64]. Specifically, it is adapted for self-radical recombination reactions, such as the $^{\cdot}$OH recombination:

$$^{\cdot}\mathrm{OH} + {}^{\cdot}\mathrm{OH} \longrightarrow \mathrm{H_2O_2} \tag{6}$$

Assuming two species A and B (Eq. 7), the reaction rate in terms of the species concentration is given by the rate equation (Eq. 8). For the self-radical recombination reactions a factor 2 has to be considered, as shown in Eq. 9.

$$\mathrm{A} + \mathrm{B} \longrightarrow \mathrm{Products} \tag{7}$$

$$-\frac{d[\mathrm{A}]}{dt} = -\frac{d[\mathrm{B}]}{dt} = k_{obs}\,[\mathrm{A}]\,[\mathrm{B}] \tag{8}$$

$$-\frac{d[^{\cdot}\mathrm{OH}]}{dt} = 2\,k_{obs}\,[^{\cdot}\mathrm{OH}]^2 \tag{9}$$

The rate at which the reaction takes place is denoted as k_{obs}. It is measured experimentally and it has two components: the intrinsic rate k_{react} and the diffusion rate k_{diff}. The three are related by the Noyes equation:

$$\frac{1}{k_{\mathrm{obs}}} = \frac{1}{k_{\mathrm{react}}} + \frac{1}{k_{\mathrm{diff}}} \tag{10}$$

For diffusion-controlled reactions, the species react immediately upon encounter when their mutual distance r is smaller than the reaction radius R. For these type of reactions, $k_{obs} = k_{diff}$. Following, it is described the procedure to link k_{diff} and R. Initially, consider the probability density function (Eq. 11) of B species diffusing towards A species, where $[\mathrm{B}]_0$ is the initial concentration of B and $\mathcal{D}_{\mathrm{AB}} = \mathcal{D}_{\mathrm{A}} + \mathcal{D}_{\mathrm{B}}$[64]. The initial conditions specify that $p(r,0) = 1$ if $r > R$ and $p(r,0) = 0$ if $r \leq R$, indicating that initially A and B have not yet

reacted. The boundary conditions are defined such that $p(r \to \infty, t) = 1$ and $p(r \leq R, t) = 0$, implying that A and B react if they encounter at a distance below R.

$$p(r,t) = \frac{[\mathrm{B}](\mathrm{r})}{[\mathrm{B}]_0} = 1 - \frac{\mathcal{R}}{r}\,\mathrm{erfc}\left\{-\frac{r-\mathcal{R}}{\sqrt{4\,\mathcal{D}_{\mathrm{AB}}\,t}}\right\} \tag{11}$$

The current I is the number of B reactants diffusing towards A species through an area $4\pi r^2$ per unit of time. It is used to link the $p(r,t)$ with the reaction law and it can be expressed in terms of $p(r,t)$ and the flux J (Fick's first law) as follows:

$$I = 4\pi r^2 J = 4\pi r^2 \mathcal{D}_{AB} \left.\frac{\partial[\mathrm{B}]}{\partial r}\right|_{\mathcal{R}} = 4\pi r^2 \mathcal{D}_{AB} \left.\frac{\partial p(r,t)}{\partial r}\right|_{\mathcal{R}} [\mathrm{B}]_0 \tag{12}$$

$$I = 4\pi \mathcal{R}\,\mathcal{D}_{AB}\left(1 + \frac{\mathcal{R}}{\sqrt{\pi\,\mathcal{D}_{AB}\,t}}\right)[\mathrm{B}]_0 \tag{13}$$

Alternatively, I can be expressed in terms of the reaction law (Eq. 14). For self-radical recombination reactions the factor 2 must be considered, as displayed in Eq. 15.

$$I = -\frac{1}{[\mathrm{A}]}\frac{d[\mathrm{A}]}{dt} = k_{diff}\,[\mathrm{B}] \tag{14}$$

$$I = -\frac{1}{[^{\cdot}\mathrm{OH}]}\frac{d[^{\cdot}\mathrm{OH}]}{dt} = 2\,k_{diff}\,[^{\cdot}\mathrm{OH}] \tag{15}$$

By comparing Eq. 13 and Eq. 14, k_{diff} can be obtained by multiplying by N_a (Eq. 16). For self-radical recombination reactions the factor 2 is considered, as displayed in Eq. 17.

$$k_{diff}(t) = 4\pi\,\mathcal{R}\,\mathcal{D}_{\mathrm{AB}}\left(1 + \frac{\mathcal{R}}{\sqrt{\pi\,\mathcal{D}_{\mathrm{AB}}\,t}}\right)N_a \tag{16}$$

$$2\,k_{diff}(t) = 4\pi\,\mathcal{R}\,\mathcal{D}_{^{\cdot}\mathrm{OH}^{\cdot}\mathrm{OH}}\left(1 + \frac{\mathcal{R}}{\sqrt{\pi\,\mathcal{D}_{^{\cdot}\mathrm{OH}^{\cdot}\mathrm{OH}}\,t}}\right)N_a \tag{17}$$
